# Plasmapheresis with corticosteroids and antiviral: a life-saving treatment for severe cases of Covid 19

**DOI:** 10.22088/cjim.11.0.572

**Published:** 2020

**Authors:** Mohammad Bagherzade, Mahmoud Parham, Somayeh Zohali, Sedighe Molaei, Jamshid Vafaeimanesh

**Affiliations:** 1 Clinical Research Development Center, Shahid Beheshti Hospital, Qom University of Medical Sciences, Qom, Iran.; 2 Student Research Committee, School of Medicine, University of Medical Sciences, Qom, Iran; 3 Gastroenterology & Hepatology Disease Research Center, Qom University of Medical Science, Qom, Iran

**Keywords:** COVID-19, therapeutic plasma exchange, corticosteroid therapy, interferon

## Abstract

**Background:**

COVID-19 pandemic is a global concern. Unfortunately, there is no exclusive treatment for critical patients to survive. In this study, we suggest using a novel three-dimensional treatment mainly based upon immune system modulation to fix the virus chaos, through cytokine storm as the main character of COVID-19 infection scenario.

**Case Presentation:**

A young man infected by SARS-CoV-2 who suffered from respiratory arrest and loss of consciousness, underwent cardiopulmonary resuscitation and endotracheal intubation. Following ICU administration and confirmed diagnosis of COVID-19, considering critical condition of the young patient, plasmapheresis was performed once on a daily basis, three doses of interferon beta(IFN-β-1b) was injected subcutaneously every other day and dexamethasone was given at a dose of 4 mg every 8 hours along with common antiviral regimen. After 2 days, the patient was extubated and transferred from the ICU to the ward where plasmapheresis was performed 4 times daily for 4 days. Finally, after 7 days of hospitalization, the patient was discharged with a good general condition.

**Conclusion:**

We modulated the immune system through plasmapheresis to sweep out the released cytokines. Also, corticosteroid along with interferon was added to common antiviral treatments. Our data suggest that this combined method is effective for critically ill COVID-19 patients.

On December 31, 2019, Wuhan City, Capital of Central China’s Hubei Province faced with patients suffering from pneumonia of unknown origin. After a quick time which it took to spread around, the cause of this coronavirus disease 2019 (COVID-19) introduced as Novel Coronavirus(SARS-CoV-2) led to a terrible epidemic and widespread global death, so far (1). Coronavirus is a member of the Coronaviridae family which targets the respiratory tract. Novel Coronavirus can lead to mild to severe lung damage and even death. Through activating the immune system and triggering inflammatory cascades, this infection leads to destruction of pulmonary alveolar epithelium followed by respiratory distress(2). Septic shock and multiple organ failure are the other consequences as well. As yet, several approaches have been implicated to treat this disease (3). One of the most effective therapies is removing cytokines and inflammatory factors and to put it better, preventing the immune system from overreacting which seems to be the main mechanism of COVID-19 damage(4). Here we report a young man infected with 2019-nCoV who suffered from sudden respiratory arrest and loss of consciousness, then treated with a three-dimensional approach including, administering corticosteroids, interferon and plasmapheresis in addition to our previous treatment regimen i.e. hydroxychloroquine and antiretroviral medication. 

## Case presentation

On March 27, 2020, a 30 year old Iranian man who was a gardener at Shahid Beheshti Hospital in Qom City, Iran, suddenly presented with loss of consciousness. Emergency medical services (EMS) was immediately provided to the patient and was taken from the hospital grounds into the emergency room. At this time, he suffered from severe respiratory distress and apnea. Accordingly, he underwent cardiopulmonary resuscitation, then intubation and followed transfer to the intensive care unit (ICU). At this time, patient’s blood oxygen saturation was 89% with FIO2 100%. Checking the vital signs, body temperature was 37/8°C and he had heart rate of 102 beats per minute, and blood pressure of 164/108 mmHg, after receiving a vasopressor (norepinephrine 5 µg/min).

Complete blood count revealed leukocytosis (WBC: 29.6×10^9/l^; lymphocyte percentage: 1.6%). Also, C-reactive protein (CRP) was 25 mg/l. VBG data of PH: 6.99, pCO_2_: 77 mmHg and HCO3:18.6 m mol/l indicated mixed respiratory and metabolic acidosis. Also, the level of IL-6 was measured 75.6 Pg/ml, which increased in comparison with normal range (0-16.4 Pg/ml).

Due to the sudden loss of consciousness, a brain computed tomography (CT) was performed to check the condition of the brain which indicated no pathological concern. Also, the patient was admitted to the cardiac service with suspicion of heart problems and cardiac examinations such as ECG and echocardiography were performed, which indicated no heart valve disease and EF of 55-60 percent.

**Figure1:**
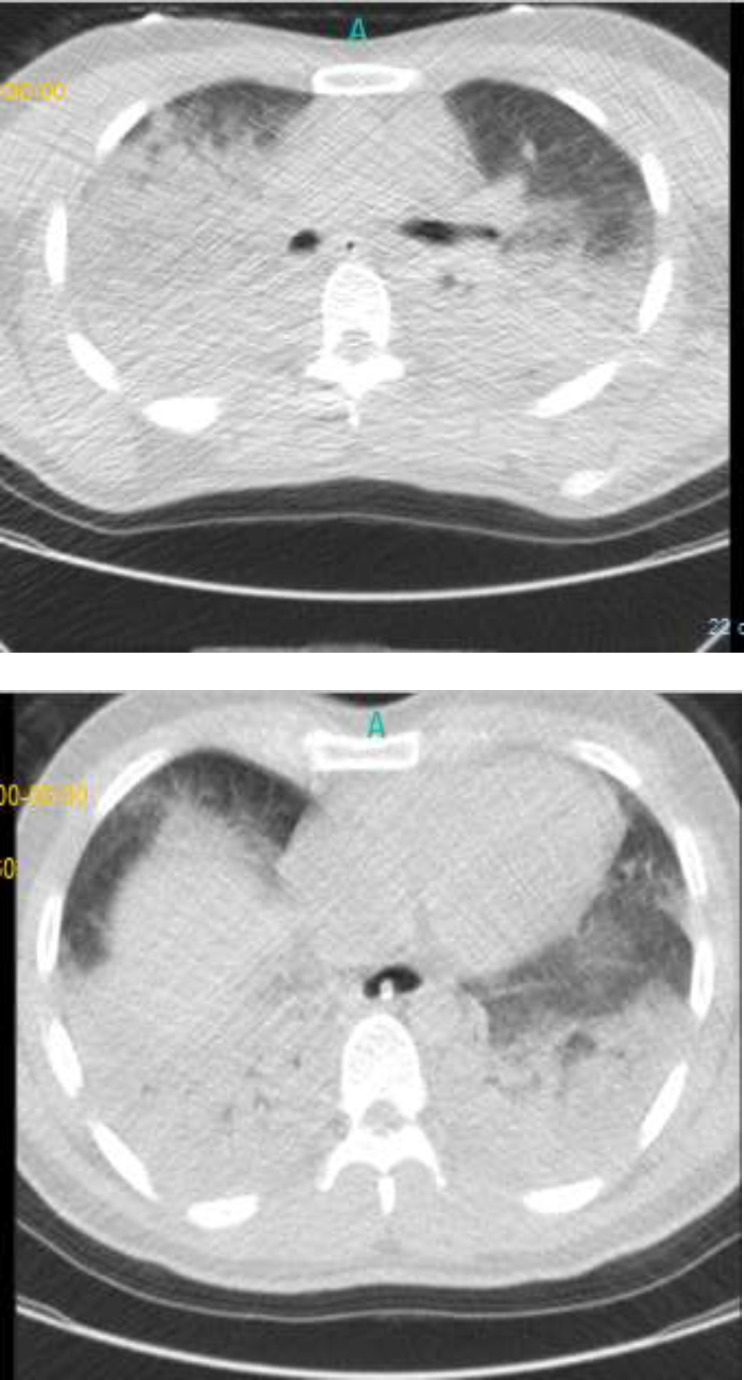
Chest CT-scans of case-patient with COVID-19 on March 27, 2020 at the onset of the disease

As the incident occurred at the epidemic zone for covid-19, detecting viral nucleic acid using qRT-PCR assay of throat swab sample and chest CT scan were considered. Images reported bilateral ground glass opacities (figure 1) and the subsequent positive viral nucleic acid PCR test, 24 hours later. During the hospitalization and after regaining consciousness, the patient was given a history of no symptoms such as cough, fever (≥38°C), chills, sore throat, runny nose, shortness of breath/difficulty in breathing , nausea/vomiting, headache and general weakness which may represent that the symptoms were not severe enough for him to notice. Also, he had no history of past underlying medical problems, medication and smoking.

Immediately after imaging and subsequent diagnosis of COVID-19, a single-dose of 400 mg hydroxychloroquine sulfate and antiretroviral medication, KALETRA® (lopinavir/Ritonavir) 100/400mg Bid were prescribed for 5 days. Considering the critical condition of the young patient, plasmapheresis was performed once on a daily basis, three doses of 250 μg interferon beta (IFN-β-1b, Ziferon®) were injected subcutaneously every other day and dexamethasone was given at a dose of 4 mg every 8 hours (TID). After 2 days, the patient was extubated and transferred from the ICU to the ward (March 31, 2020), where plasmapheresis was performed 4 times daily for 4 days.

**Figure2:**
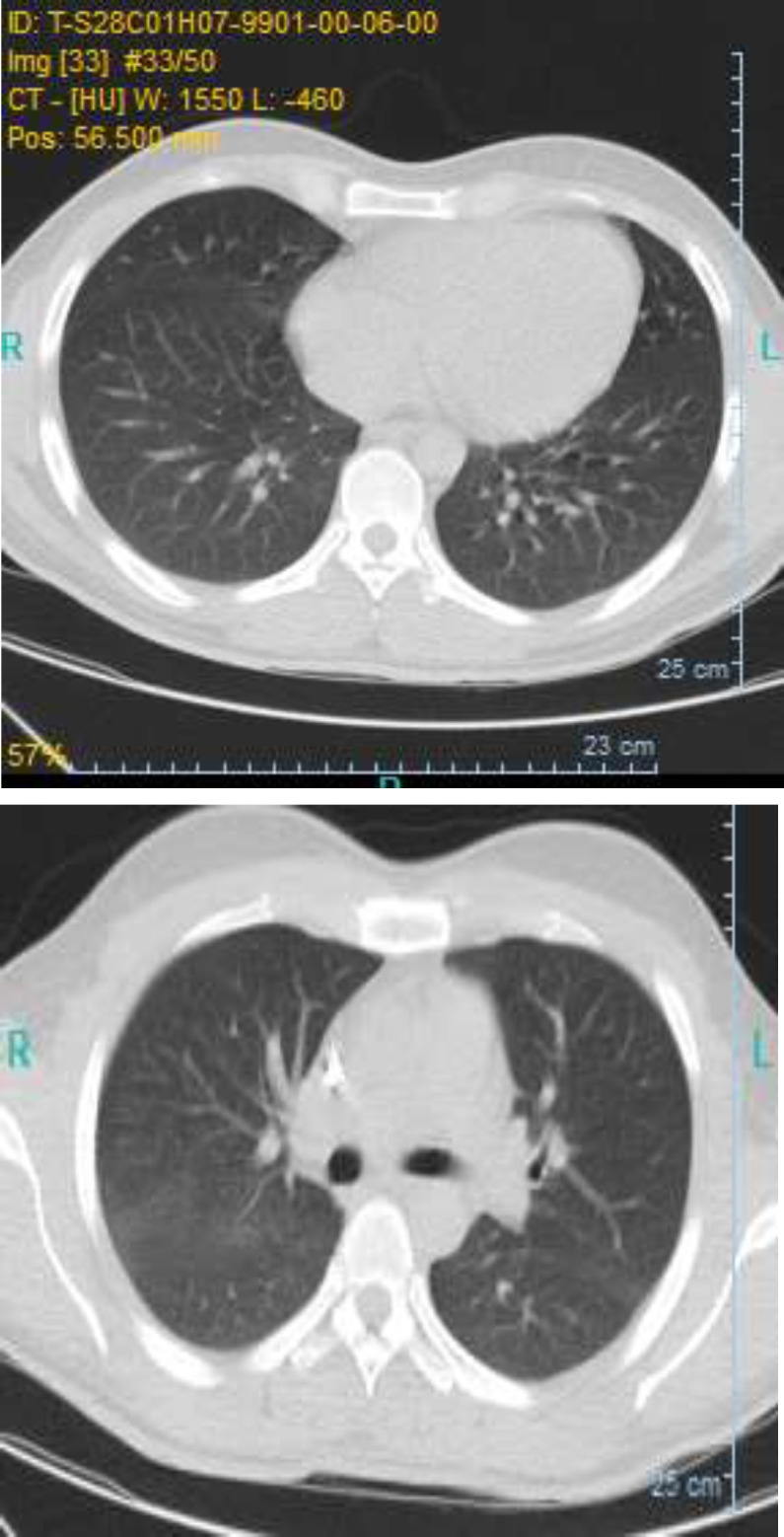
Chest CT-scans of case-patient with COVID-19 on April 2,2020after recovery

Finally after 7 days of hospitalization, the patient was discharged with a good general condition and 97% oxygen saturation (without oxygen mask) with full consciousness (April 2, 2020). On the last day of hospitalization, chest CT scan was taken again and the findings confirmed patient´s recovery upon resolution of pulmonary infiltrates compared to previous images (figure 2). On the same day, the results of his complete blood count were as follows: WBC: 14.3×10^9/l^; lymphocyte percentage: 6.3%.Also, CRP was 25 mg/l.

## Discussion

Coronavirus disease severity has a wide spectrum from mild presentations or even asymptomatic carriers to death (5).It has been suggested that the main pathogenesis of COVID-19 as a respiratory virus is overproduction of inflammatory cytokines, called cytokine storm syndrome which results in acute lung damage (6). As a result of extensive inflammation and increased vascular permeability, acute lung damage may occur. Studies have found that in severe cases of COVID-19 admitted to ICU, high levels of inflammatory factors such as, IL-2, IL-6, IL-7, IL-10, GCSF, IP10, MCP1, MIP1α and TNF-α were detected, which could be a reason for rapid disease progression(7). Upon cytokine release, alveolar macrophages and neutrophils are activated, resulting in alveolar epithelium and vascular endothelium destruction (8). The level of IL-6 was clearly higher in our patients compared to normal ranges. One of the most important cytokines in cytokine release syndrome is IL-6 which aggravates the patients clinical condition by causing vascular leakage, complement activation and coagulation cascade imbalance along with cardiomyopathy damaging myocardial cells (6). 

Being suggested as a COVID-19 severity predictor, IL-6 inhibitors may reduce the cytokine storm syndrome, especially in critically ill patients (9, 10). Here we used IFN-β-1b which shows most of its systematic effects through IL-6 modulation (11) to alleviate COVID-19 induced cytokine damage. According to these findings, removing pro-inflammatory mediators, such as cytokines, immune complexes and antibodies through plasma exchange seems to be effective, especially in severe forms of disease. Data in line with our research suggest that therapeutic plasma exchange in COVID-19 patients with sepsis and multiple organ failure reduces mortality by alleviating cytokine storm and inflammation (12). Also, it could reduce virus load in the body. Though combined with common treatment regimen for COVID-19 in Iran i.e. hydroxychloroquine(13)and antiretroviral medication, lopinavir/ritonavir as protease inhibitors typically used in HIV to which block viral replication (14), plasmapheresis could be a very efficient method against COVID-19 damage. On the other hand, corticosteroids, the anti-inflammatory hormones used in 2003 SARS epidemic as the mainstay of immunosuppressive therapy to improve the patient's condition leading to their initial recovery (15) showed effective help in COVID-19 critical patients. Also, a recent clinical trial has suggested that the early administration of glucocorticoids, in the form of dexamethasone, effectively alleviated the pulmonary inflammatory reactions in acute respiratory distress syndrome (ARDS) and reduced mechanical ventilation duration and mortality Among the patients with ARDS upon COVID-19 infection in Wuhan, China, administering methylprednisolone decreased mortality rate (16). 

A retrospective study on 244 critically ill patients with COVID-19 showed lower dosage of corticosteroids with limited time could help the survival rate, in contrast with higher dosages which increased mortality(17). Another study suggests that dexamethasone for early administration in moderate to severe ARDS patients reduces mechanical ventilation duration(18). Although, some studies have shown the primary treatment with corticosteroids in patients with SARS-CoV infection may worsen their symptoms by increasing the virus load (19, 20)which prolonged use of glucocorticoids may found to be guilty, considering reported side effects.

In that case, using a target-based immunomodulatory lever may seem as an efficient tool against COVID-19 infection. In a typical scenario, a virus-infected cell will release interferons causing nearby cells to heighten their anti-viral defenses. Secretion by various cell types, notably plasmacytoid dendritic cells and binding to a specific cell surface receptor complex known as the IFN-α/β receptor (IFNAR), activates antiviral genes to control innate and adaptive immunity and intracellular antimicrobial program. In a recent study, SARS-CoV-2 has introduced as the most sensitive of all Coronaviruses to interferon and IFN-β subtype seems to be the most effective one on COVID-19(21). Interferon alfa-2b is likely to be one of the most promising drugs used against this epidemic in China(22)and other following countries. The underlying condition may be due to functional exhaustion of antiviral lymphocytes as is suggested by some research at the early stage of COVID-19 infection (23). However, there are research studies suggesting that the JAK inhibitor baricitinib, with IFN-blocking activity might ameliorate COVID-19 symptoms(24)which even took a step further, suggesting combining antiviral and anti-inflammatory treatments, although considering concerns over virus remaining and reactivation later on (25).

In conclusion, we are suggesting a novel combination treatment for the coronavirus disease (COVID-19) using immunosuppressive scalepan through short-term corticosteroid therapy and also plasmapheresis to sweep out the released cytokines along with antiviral treatments by lopinavir/Ritonavir in additional to direct antiviral immunomodulation through interferon as the other pan which enabled us to change a patient status from life-threatening condition to recovery state. However, there is a need for high scale randomized clinical trial with more patients for this work to be considered as a combinational approach toward critically ill COVID-19 patients.
